# Predicting CT-Based Coronary Artery Disease Using Vascular Biomarkers Derived from Fundus Photographs with a Graph Convolutional Neural Network

**DOI:** 10.3390/diagnostics12061390

**Published:** 2022-06-04

**Authors:** Fan Huang, Jie Lian, Kei-Shing Ng, Kendrick Shih, Varut Vardhanabhuti

**Affiliations:** 1 Department of Diagnostic Radiology, LKS Faculty of Medicine, The University of Hong Kong, Hong Kong, China; fhuang@hku.hk (F.H.); jlian@connect.hku.hk (J.L.); dougng@hku.hk (K.-S.N.); 2 Department of Ophthalmology, LKS Faculty of Medicine, The University of Hong Kong, Hong Kong, China; kcshih@hku.hk

**Keywords:** CAD-RADS, coronary artery disease, fundoscopy, fundus image analysis, graph convolutional neural network

## Abstract

The study population contains 145 patients who were prospectively recruited for coronary CT angiography (CCTA) and fundoscopy. This study first examined the association between retinal vascular changes and the Coronary Artery Disease Reporting and Data System (CAD-RADS) as assessed on CCTA. Then, we developed a graph neural network (GNN) model for predicting the CAD-RADS as a proxy for coronary artery disease. The CCTA scans were stratified by CAD-RADS scores by expert readers, and the vascular biomarkers were extracted from their fundus images. Association analyses of CAD-RADS scores were performed with patient characteristics, retinal diseases, and quantitative vascular biomarkers. Finally, a GNN model was constructed for the task of predicting the CAD-RADS score compared to traditional machine learning (ML) models. The experimental results showed that a few retinal vascular biomarkers were significantly associated with adverse CAD-RADS scores, which were mainly pertaining to arterial width, arterial angle, venous angle, and fractal dimensions. Additionally, the GNN model achieved a sensitivity, specificity, accuracy and area under the curve of 0.711, 0.697, 0.704 and 0.739, respectively. This performance outperformed the same evaluation metrics obtained from the traditional ML models (*p* < 0.05). The data suggested that retinal vasculature could be a potential biomarker for atherosclerosis in the coronary artery and that the GNN model could be utilized for accurate prediction.

## 1. Introduction

Atherosclerosis is a chronic inflammatory disease of the arteries which is due to the buildup of plaques adhering to the inner vessel wall. The early detection of atherosclerosis is crucial for early treatment and prevention. However, current clinical diagnosis techniques, such as coronary computed tomography angiography, tend to only identify the plaques at their advanced stages rather than in the early stages. More importantly, medical imaging is usually utilized when clear symptoms of atherosclerosis, such as acute chest pain, are observed in high-risk patients. Early subclinical disease detection remains a challenge. Hence, finding additional variables for the risk stratification or even the early detection of atherosclerosis is needed.

Changes in the micro-vasculature, such as vessel nicking/narrowing, have been recognized as early indicators for macro-vascular abnormalities. It was believed that the common pathophysiologic processes of atherosclerosis may underlie both macro-vascular and micro-vascular disease [1,2,3].

The retinal vasculature shares similar anatomical and physiological characteristics with the coronary circulations [4,5,6]. It can be non-invasively acquired by a fundus camera and quantitatively measured on the digital fundus images using machine learning techniques [7]. Several recent studies have focused on investigating the association between retinal microvascular changes and the risk factors for cardiovascular diseases. Klein et al. [8] examined the relationships of retinal arteriolar changes with clinical and subclinical manifestations of atherosclerosis. They found that the arteriolar-to-venular ratio (A/V ratio), after adjusting for present and previous blood pressure and medications, was associated with the presence of carotid plaque. Hubbard et al. [9] suggested that a lower A/V ratio was associated with increased blood pressure and incidence of cardiovascular diseases independently of other known risk factors (e.g., gender, age, blood pressure). Ikram et al. found that the venular diameters were linearly related to several markers of atherosclerosis (e.g., leukocyte count, erythrocyte sedimentation rate, HDL levels etc.), and a lower AVR was significantly related to a higher carotid plaque score [10]. Lyu et al. showed that the occurrence of retinal vein occlusion was significantly associated with increased LDL cholesterol levels, increased brachial-ankle pulse wave velocity (baPWV)and the presence of carotid plaques [11]. Wong et al. studied the association of retinopathy (e.g., the presence of microaneurysms, hemorrhages, cotton wool spots, hard exudates and etc.) with coronary artery calcification scores as on cardiac computed tomography [12].

The Coronary Artery Disease Reporting and Data System (CAD-RADS) was proposed in 2016 and soon became the standardized reporting system for coronary artery disease for the outpatient, inpatient and emergency department settings [13]. It assesses the stenosis severity of the coronary arteries on coronary computed tomography angiography (CCTA) and categorizes the severity into 6 groups: 0 (0%, normal), 1 (1–24%, minimal) and 2 (25–49%, mild), 3 (50–69%, moderate), 4 (70–99%, severe) and 5 (100%, occluded). Based on these scores, recommendations for subsequent management have been proposed allowing standardization in medical practices.

Being motivated by previous studies on retinal vascular changes and atherosclerosis, it is conceivable that the quantitative retinal vascular biomarkers can be used to predict coronary artery disease using CAD-RADS as a proxy for cardiovascular disease. In this study, we first examined the association between retinal vascular changes and CAD-RADS. Then, we utilize a graph convolutional neural network (GNN) model to predict the CAD-RADS (assessed on CCTA) using the quantitative vascular biomarkers (derived from fundus images) of the same subjects.

## 2. Materials and Methods

**Figure 1:**
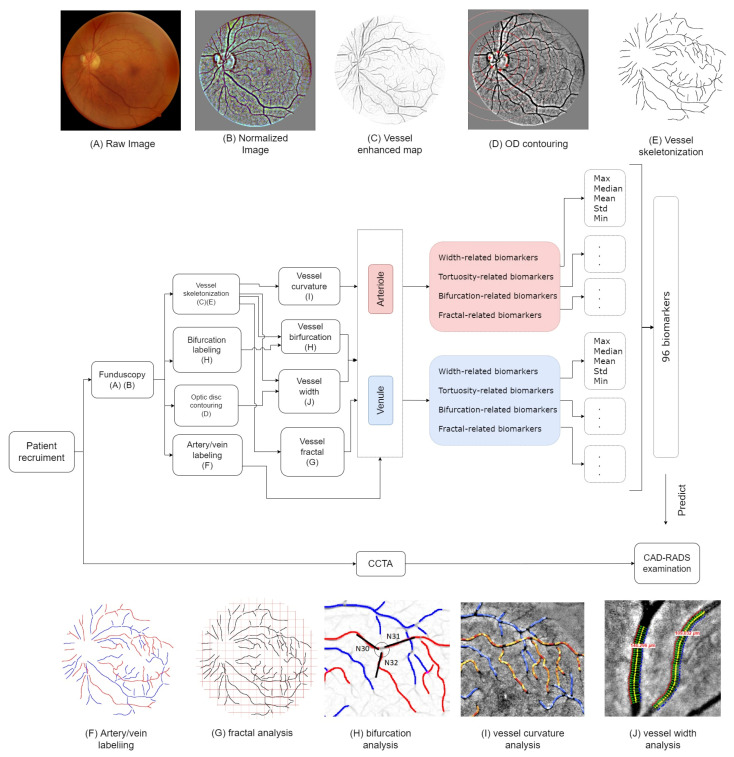
The pipeline diagram for this study. Patients were recruited for CCTA and fundus eye examinations on the same day. The CCTA scans were diagnosed and stratified based on the CAD-RADS guideline. (**A**,**B**): The fundus images were acquired and pre-processed by brightness normalization. (**C**,**E**): The blood vessel centerlines were manually annotated, and the bifurcation relationship at each junction was labeled. (**D**): The optic disc was contoured, and the optic disc diameter was measured. (**F**): The type of the vessels was categorized into arteries or veins. (**G**–**J**): Multiple biomarkers on the extracted vasculature, including the vessel curvature, bifurcation feature, width, and the fractal for arteries and veins, were extracted, respectively. We measured the maximum, mean, median and minimum value for each biomarker, which yielded, in total, 96 biomarkers for each fundus image. At last, we built machine learning models to predict the corresponding CAD-RADS score.

This prospective single-centre study included patients from a tertiary hospital in Hong Kong from May to October 2019. This study was approved by the local institutional review board. All subjects gave full written consent for the study. The patients received both fundoscopy examination and coronary computed tomography angiography on the same day. The CAD-RADS scores for the patients were stratified based on their CCTA scans. Their fundus images were processed to extract, in total, 96 vascular biomarkers (detailed below). A diagram shown in Figure 1 summarizes the pipeline of this study.

### 2.1. Study Population

In this study, we enrolled 173 subjects who were ambulatory patients scheduled for outpatient radiological investigations (i.e., coronary computed tomography angiography). The subjects were recruited on the same day as their CCTA scanning for a fundoscopy examination. Patient demographics, clinical history, and comorbidities were recorded. Blood pressure and heart rate were measured at the time of enrollment.

### 2.2. Fundus Examination

The fundoscopy image acquisition was performed on-site in a dedicated room. A color, non-stereo and non-mydriatic fundus camera, FundusVue (Crystalvue, Taoyuan City, Taiwan), was used for the image acquisition. The acquired image size was 2592 × 1944 pixels, and all images were stored in JPEG compression format. Examinations were performed in ambient lighting with a dark cloth drape over the participants’ heads during image acquisition. During the examination, macula-centered, 45° field-of-view retinal fundus photographs were taken for both left and right eyes. The duration of a single screening took 3–5 min.

### 2.3. Grading of Fundoscopy Images and Subject Exclusion

**Figure 2:**
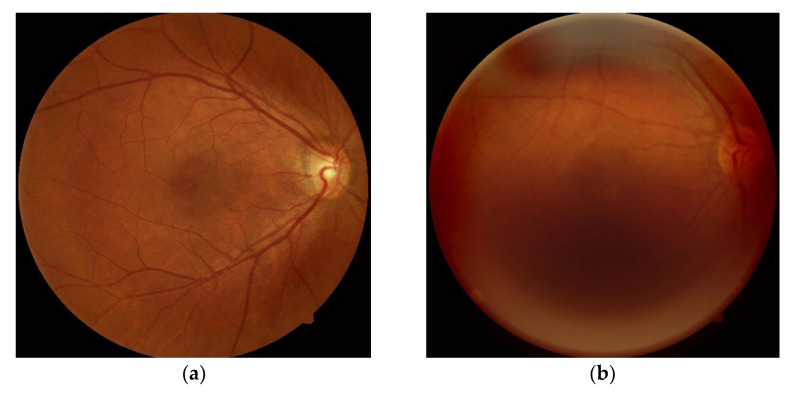
The image quality of fundoscopy was graded by two ophthalmologists. The fundus images with sub-optimal quality were excluded in this study. (**a**) Satisfactory image; (**b**) sub-optimal image.

To analyze the association between retinopathy and the CAD-RADS scores, all acquired fundus images were independently reviewed by two clinical ophthalmologists from our institution in Hong Kong with 10 and 12 years of experience in fundus image interpretation. They scored for the presence or absence of four common eye diseases: (1) tessellated retina (TR), (2) DM-related retinopathy (DM-R); (3) age-related macular degeneration (AMD) and (4) pathologic myopia (PM). The readers also graded the image quality (satisfactory/sub-optimal) during the reading. Figure 2 shows an example of satisfactory and sub-optimal images, respectively. If both left and right eye fundus images were of poor quality, the patient was excluded from the study. Consequently, 28 subjects were excluded from the study. The demographics of the remained study population are summarized in Table 1.

### 2.4. Coronary Computed Tomography Angiography (CCTA) Acquisition and Examination

Coronary CT scans were performed on a 320 MDCT scanner (Aquilion One, Canon Medical System, Tokyo, Japan). All patients underwent a standardized protocol of pre-medication with oral and intravenous beta-blockers for lowering heart rate to <65 bpm if needed. Sublingual glyceryl trinitrate (GTN) spray was given prior to scan acquisition. Prospective ECG gating was used. Intravenous iodinated contrast was injected during scan acquisition. The scans that were non-diagnostic were excluded from the study and were not considered further in our study.

### 2.5. CAD-RADS Score

The CAD-RADS score was developed to standardize CCTA reporting and was assessed on a per-patient basis. The CAD-RADS score represents the highest-grade coronary artery lesion documented by CCTA. It ranges from CAD-RADS 0 (zero) for the complete absence of stenosis and plaque, to CAD-RADS 5 for the presence of at least one totally occluded coronary artery [13]. The examples of CCTA for CADRADS 1 (minimal) to 5 (occluded) are shown in Supplementary Figure S1.

In this study, we examined the association between fundus vascular biomarkers and the CAD-RADS scores via binary classification. We binarized the original CAD-RADS scores (range from 0 to 5) in two ways, yielding two different predictive models. For model 1, the negative class 0 included CAD-RADS scores ≤1 (i.e., normal and minimal), and the positive class 1 included CAD-RADS scores ≥2 (i.e., mild, moderate, severe stenosis and occlusion). This model is intended to discover early signs of atherosclerosis. For model 1, the control group and the abnormal group were separated equally, where the control group (class 0) had 70 subjects, and the abnormal group (class 1) had 75 subjects. For model 2, we divided the study population into patients of non-significant CAD-RADS (CAT = 0) and significant CAD-RADS (CAT = 1). The subjects of significant CAD-RADS were of high severity of coronary artery disease. They were the patients, according to their clinical records, who were scheduled for surgical intervention, or who had prior intervention (stent or coronary artery bypass graft (CABG) found in the CCTA) or who had been stratified to CAD-RADS ≥ 4. Then the remainder were regarded as non-significant CAD-RADS.

### 2.6. Fundus Biomarkers

In this study, we focus on the quantitative biomarkers of the retinal microvasculature, which can be summarized into four main categories: vessel width, vessel tortuosity, vessel junction property and vessel fractal dimensions. A diagram is shown in Figure 1. A total of 96 biomarkers were generated.

### 2.7. Vessel Manual Modelling

Retinal vessel skeletonization is an essential step for the quantitative analysis of retinal vessel structures. In this study, the vasculature was manually modelled on a vessel-enhanced map of the fundus images.

First of all, the green channel of the color images was extracted and brightness-normalized as it provided the best contrast for the vasculature [7]. Afterwards, the vessel enhancing method proposed by Zhang et al. was employed to obtain the vessel probability map (named soft segmentation) [14]. This technique is based on applying a set of multi-scale left-invariant derivative (LID) filters and locally adaptive derivative (LAD) filters on the orientation scores of an image. It robustly enhances elongated structures (i.e., the vasculature) and suppresses the background structure, yielding the enhancement of a complete retinal vessel network.

**Figure 3:**
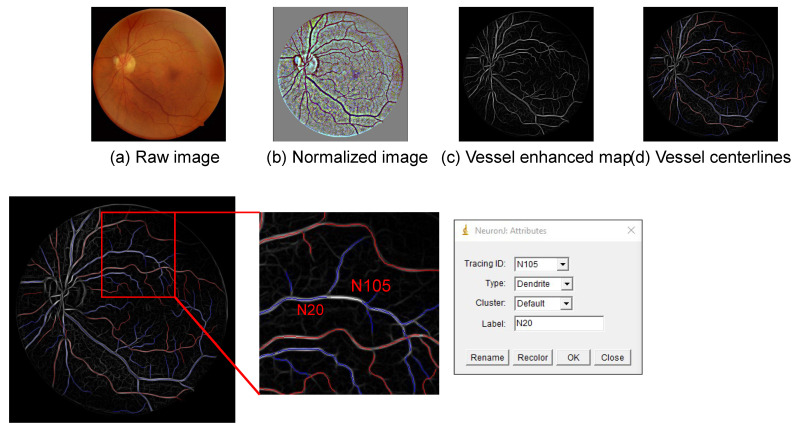
The retinal vasculature was manually annotated by using the “NeuronJ” plugin in ImageJ software. The vessel centerlines were traced on the vessel soft segmentation map. The parent-child relationship of the vessels was determined using the labelling system in “NeuronJ”.

Afterwards, we used the open-source Java plugin “NeuronJ” for ImageJ (National Institutes of Health, Bethesda, MD, USA) to manually model the retinal vasculature on the vessel enhanced map [15]. First of all, we traced the centerline for each vessel segment on the image with branching points broken. Then, we determined the vessel type (i.e., artery/vein) using the default names for neurons, i.e., axon for arteries and dendrite for the veins (as shown in Figure 3). At last, we labelled parent-child relationships for the blood vessels using the labelling system of “NeuronJ”. If a vessel segment originated from the optic disc or its parent was not presented in the image, we labelled its parent as “N0”.

### 2.8. Optic Disc Labelling

The optic disc is an important landmark for retinal image analysis. In this study, we obtained the boundary of the optic disc by manually clicking 6 points and fitted a rotated ellipse to these points. Afterwards, the pixel size for the image was estimated by taking the ratio between the general optic disc diameter (1800 µm) and the longest diameter (in pixel) [16]. The pixel size was used to convert the vessel width measured in pixels to the actual width in µm.

### 2.9. Vessel Width

Many clinical studies have shown that the changes in retinal vessel caliber are associated with the progress of a variety of systemic diseases [5,17,18,19]. In this study, we measured the vessel width using the method proposed by Huang et al. [20,21]. In brief, this technique utilizes the geodesic active contour model proposed by Caselles et al. [22]. For every vessel segment, it initializes an enclosed contour by expanding the extracted vessel centerline. Afterwards, the contour is iteratively deformed to fit a smooth boundary over the vessel segment on the normalized green channel image. We computed the distance from one detected vessel edge to the other one for each control point on the contour. The measured distances with extreme values were eliminated to avoid outliers. The final vessel width was estimated as the average of the remained edge distances.

**Figure 4:**
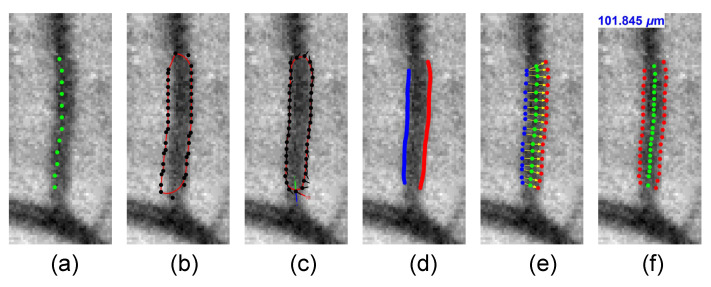
(**a**) A vessel centerline is initialized by the vessel skeletonization. (**b**) An active contour model is built based on the centerline. (**c**) The contour iteratively grows and fits the left and right boundaries of the vessel. (**d**) The contour is cropped to obtain the left and right boundary (**e**). (**f**) The vessel width is measured by finding the average distances from one side to the other.

The measured distances with extreme values were eliminated to prevent outliers, and vessel width was calculated as the average of the remaining measurements. Then the contour was evolved iteratively and fitted to the boundaries of the vessel. Finally, the vessel caliber was measured by computing the distance from one detected vessel edge to the other one. The processing pipeline for the width measurement of the vessel segments is summarized in Figure 4).

### 2.10. Vessel Tortuosity

The vessel curvature is another important biomarker of the vasculature, which is defined as the integration of curvature along a curve. In this study, we computed in total 14 curvature-based metrics for every vessel segment as summarized by Kalitzeos et al. [23]. (1) $\frac{\mathrm{Arc}\;\mathrm{length}}{\mathrm{Chord}\;\mathrm{length}}$; (2) $\frac{\mathrm{Arc}\;\mathrm{length}}{\mathrm{Chord}\;\mathrm{length}}-1$; (3) Total curvature; (4) Total squared curvature; (5) $\frac{\mathrm{Total}\;\mathrm{curvature}}{\mathrm{Arc}\;\mathrm{length}}$; (6) $\frac{\mathrm{Total}\;\mathrm{squared}\;\mathrm{curvature}}{\mathrm{Arc}\;\mathrm{length}}$; (7) $\frac{\mathrm{Total}\;\mathrm{curvature}}{\mathrm{Chord}\;\mathrm{length}}$; (8) $\frac{\mathrm{Total}\;\mathrm{squared}\;\mathrm{curvature}}{\mathrm{Chord}\;\mathrm{length}}$; (9) Integrated curvature; (10) Smooth tortuosity index; (11) Computer-Aided Image Analysis of the Retina (CAIAR) tortuosity index; (12) Tortuosity coefficient-01; (13) Tortuosity coefficient-02; and (14) Standard deviation tortuosity.

### 2.11. Bifurcation Junction Parameters

The branching pattern at vessel bifurcations might reflect pathological changes in the circulation system. It was believed that the vasculature is not a totally random network but has been developed under some optimum physical principles, e.g., the minimum friction between blood flow and vessel wall, the optimal heart rate to achieve proper blood supply and the shortest transport distances, etc. In this study, we calculated the bifurcation properties proposed by Al-Diri et al. [24]. In brief, let *d*_0_, *d*_1_ and *d*_2_ be the width of parent vessel and its daughter vessels, *θ*_1_ and *θ*_2_ be the angle between two daughter vessels and the parent vessels, and *θ* be the angle between the two daughter vessels. The bifurcation optimality can be quantitatively measured by various metrics, including: (1) the asymmetry ratio $\alpha ={\left(\frac{d_{2}}{d_{1}}\right)}^{2}$; (2) the area-ratio $\beta =\frac{d_{1}^{2}+d_{2}^{2}}{d_{0}}$; (3) the bifurcation index $\lambda =\frac{d_{2}}{d_{1}}$; (4) diameter ratio $\lambda_{1}=\frac{d_{1}}{d_{0}}$ and $\lambda_{2}=\frac{d_{2}}{d_{0}}$; (5) the bifurcation angle *θ*, *θ*_1_ and *θ*_2_, where *θ* = *θ*_1_ + *θ*_2_.

### 2.12. Vessel Fractal Dimensions

The last vascular biomarker that describes the overall vascular changes is the fractal dimension. The fractal dimension measures the general complexity of a self-similar structure, i.e., the vascular tree. In this study, we computed three fractal methods that are widely used in the literature for the vasculature (the centerlines): (1) the box dimension DB; (2) the information dimension DI; and (3) the correlation dimension (DC) [25,26,27].

### 2.13. The GraphSAGE Model

Applying graph analysis to population data for disease prediction is highly beneficial [28]. A population graph is constructed based on two important elements: nodes and edges, where the nodes are the individuals from a population pool, and the edges are the user-defined linkage/similarity between every two persons. In this study, we built our population graph at an image level, where the graph nodes represent the fundus image samples of the left/right eye of the participants. The nodes are characterized by the quantitative vascular biomarkers as introduced above. For the edges, we defined a similarity score calculated based on the age and gender difference between every two participants. In brief, if two subjects have the same gender and their age difference was less than 5 years old, their similarity score was 2. If either one of the criteria matched, the similarity score was 1. If none of the criteria matched, the similarity score was 0, and the edge between their nodes was removed. The detail of the similarity score is summarized in Appendix A.

**Figure 5:**
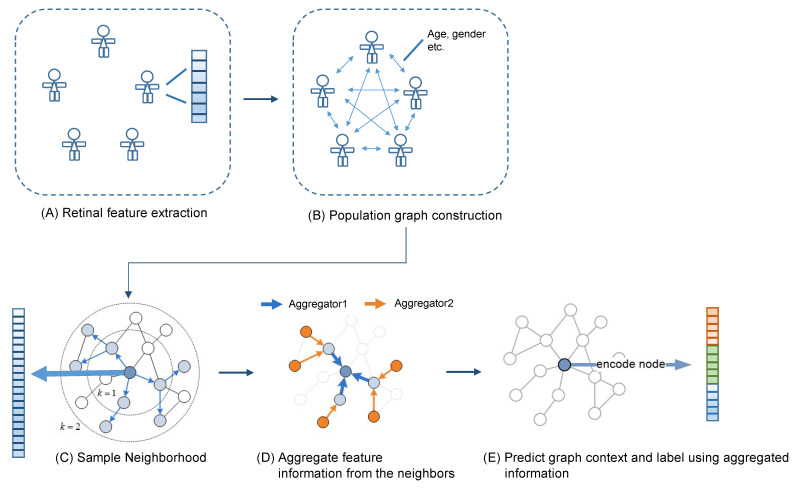
(**A**–**E**) The population graph was constructed in which the nodes represent the fundus images characterized by 96 retinal vascular biomarkers, and the edges were the similarity score determined by the age and gender of the subjects. A GraphSAGE network was applied to the constructed graph to predict CAD-RADS score of the subjects.

After the graph was constructed, we trained a graph convolutional neural network for the CAD-RADS prediction. In this study, we utilized the graph sample and aggregate network (GraphSAGE) network for the CAD-RADS score prediction [29]. It is a graph neural network framework developed for inductive representation learning on large graphs, which have rich node attributes such as high-dimensional node features and complicated connectivity between the nodes. During the training, the network learns aggregation functions that generate low-dimensional vectors from the embedding of the high-dimensional node features and the complicated node connectivity. We used a two-layer architecture with a sum-readout layer as the prediction model. The graph construction and the GNN model were summarized in Figure 5. The network architecture is summarized in Appendix B.

### 2.14. Traditional Machine Learning Models

Besides the GraphSAGE model, we also examined the five most commonly used machine learning models on the same dataset for comparison. We tested the logistic regression classifier (LR), the linear discriminant analysis classifier (LDA), the k-nearest neighbour classifier (kNN), the naïve Bayes classifier (NB) and the support vector machine classifier (SVM). All these classifiers were built-in modules in the scikit-learn (version 1.0.1) python package [30].

### 2.15. Feature Selection and Dimensionality Reduction

Feature reduction was performed prior to training the GCN model and the machine learning classifiers. In this study, we examined various feature selection techniques as summarized by Li et al. [31]. In this paper, we selected the best feature selection technique for the CAD-RADS models, respectively, i.e., giving the best area under the curve (AUC) in a repeated 10-folds cross-validation and reported the corresponding classification performances. The final selected feature selection techniques include: (1) correlation-based feature selection (CFS) [32]; (2) conditional mutual information maximization (CMIM) [33]; (3) double input symmetrical relevance (DISR) [34]; (4) interaction capping (ICAP) [35]; (5) Laplacian score-based feature selection (LAP) [36]; (6) support vector machine backward (SVMB) and (7) no feature selection used (all).

### 2.16. Statistical Study

We examined the odds ratio of the CAD-RADS scores to the presence of the four eye diseases, diagnosed by the ophthalmologists, by multinomial logistic regression analysis using SPSS 26 [37]. In the multinomial logistic regression analysis, we firstly adjusted for age and gender (denoted as OR-model 1) and additionally adjusted for cardiovascular risk factors, including systolic and diastolic blood pressure, heart rate, body mass index (BMI), diabetes stage and current cigarette smoking (denoted as OR-model 2).

To evaluate the performance of the machine learning classifiers, we applied a repeated 10 folds cross-validation procedure and computed the average of sensitivity (Sens.), specificity (Spec.), accuracy (Accu.), the receiver operating characteristic (ROC) curve and its area under the curve (AUC), F_1_-scores and precision. We measured these metrics based on image-wise vs. subject-wise levels. The subject-wise results were obtained by averaging the left and right eye predictions (if both eyes were available). A McNemar’s test was used to assess the statistical difference between the GraphSAGE model and the traditional machine learning models.

We assessed the importance of each feature by examining its odds ratios and the *p*-value (under 95% confidence interval) among the CAD-RADS scores of multinomial logistic regression analysis. *p* < 0.05 indicates that the feature is significantly associated with the CADRADS score.

## 3. Results

A total of 145 subjects were included in the study, of which 78 subjects had both the eyes included and 67 subjects had only one eye included (either the left or the right eye). Characteristics of the study population across model 1 and model 2 are shown in Table 1 and Table 2.

### 3.1. Association Analysis of CAD-RADS Scores with Patient Characteristics, Retinal Diseases, and Quantitative Vascular Biomarkers

For the CAD-RADS model 1 CAD-RADS ≤ 1, there were 25/70 (35.71%) subjects who had retinopathy. Of these, 21.43%, 2.86%, 11.43% and 1.43% of subjects were diagnosed with tessellated retina, DM-related retinopathy, AMD and pathologic myopia, respectively. For CAD-RADS model 1 CAD-RADS ≥ 2, there were 32/75 (42.67%) subjects who had retinopathy. Of these, 24%, 5.33%, 12% and 4% of subjects were diagnosed with tessellated retina, DM-related retinopathy, AMD and pathologic myopia, respectively. Subjects with CAD-RADS ≥ 2 were significantly older than subjects with CAD-RADS ≤ 1 (Mann–Whitney U test, *p* < 0.01), with higher BMI (*t*-test, *p* = 0.064), lower heart rate (Mann–Whitney U test, *p* = 0.059), and more male than female (Chi-square test, *p* < 0.05). No differences were found in terms of blood pressure (both the systolic and diastolic pressure), tobacco usage, and education level via Chi-square testing.

For the CAD-RADS model 2 non-significant CAD-RADS group, there were 44/108 (40.74%) subjects who had retinopathy. Of these, 25%, 3.7%, 12.04% and 3.7% of subjects were diagnosed with tessellated retina, DM-related retinopathy, AMD and pathologic myopia, respectively. For the OR-model 2 significant CAD-RADS group, there were 13/37 (35.14%) subjects who had retinopathy. Of these, 16.22%, 5.41%, and 10.81% were diagnosed with tessellated retina, DM-related retinopathy, and AMD, respectively. The differences in terms of age and BMI for CAD-RADS-S = 0 and CAD- RADS-S = 1 were not significant (Mann–Whitney test, *p* = 0.32 and *p* = 0.87, respectively). Subjects of CAT = 1, compared to those of CAT = 0, generally had lower heart rates (Mann–Whitney test, *p* = 0.054) and were more likely to be male (Chi-square test, *p* < 0.05). No differences were found in terms of blood pressure (both the systolic and diastolic pressure), tobacco usage and education level via Chi-square testing.

In the multinomial logistic regression shown in Table 2, after adjusting for age, gender (OR-model 1), and additionally adjusting for cardiovascular risk factors (OR-model 2), we found no associations between the two CAD-RAD models and the ophthalmologist-diagnosed retinopathy (see Supplementary Table S1). In Supplementary Table S2, we summarized the results between two CAD-RADS models and each of the extracted retinal vascular biomarkers. For CAD-RADS model 1, it was found that a few retinal vascular biomarkers were significantly associated with adverse CAD-RADS scores. These were mainly pertaining to arterial width, arterial angle, venous angle, and features relating to fractal dimensions.

### 3.2. GNN and Traditional Machine Learning Models

Table 3 summarises the classification results of the CAD-RADS GNN models (model 1 and model 2). In this study, we reported the performance of the model in terms of image-wise level and subject-wise level.

For the image-wise prediction on CAD-RADS model 1, the GraphSAGE network, using all the vascular biomarkers, achieved 0.711 (95%CI: (0.621, 0.786)) for sensitivity, 0.697 (95%CI: (0.605, 0776)) for specificity, 0.704 (95%CI: (0.644, 0.764)) for accuracy, 0.739 (95%CI: (0.675, 0.804)) for the AUC, 0.711 (95%CI: (0.672, 0.746)) for F_1_-score and 0.711 (95%CI: (0.621, 0.786)) for precision. The SVM model, using all the extracted features, achieved an AUC of 0.604 (95%CI: (0.53, 0.678)). Other traditional machine learning models, using either CFS or DISR feature selection methods, achieved AUCs range from 0.507 to 0.527.

For the subject-wise prediction on CAD-RADS model 1, the GraphSAGE network, with the features selected by the LAP method achieved 0.747 (95%CI: (0.638, 0.831)) for sensitivity, 0.571 (95%CI: (0.455, 0.681)) for specificity, 0.662 (95%CI: (0.585, 0.739)) for accuracy, 0.769 (95%CI: (0.708, 0.831)) for AUC, 0.725 (95%CI: (0.679, 0.768)) for F_1_-score and 0.651 (95%CI: (0.546, 0.743)) for precision. The SVM model, with the features selected by the SVMB method, achieved an AUC of 0.697 (95%CI: (0.629, 0.765)). The remaining machine learning models obtained AUCs ranging from 0.492 to 0.531.

For the image-wise prediction on CAD-RADS model 2, the GraphSAGE network, using all the vascular biomarkers, achieved 0.544 (95%CI: (0.416, 0.666)) for sensitivity, 0.681 (95%CI: (0.606, 0.747)) for specificity, 0.646 (95%CI: (0.583, 0.709)) for accuracy, 0.692 (95%CI: (0.608, 0.776)) for the AUC, 0.497 (95%CI: (0.442, 0.552)) for F_1_-score and 0.369 (95%CI: (0.274, 0.476)) for precision. The other prediction models achieved AUCs ranging from 0.497 to 0.561.

For the subject-wise prediction on CAD-RADS model 2, the GraphSAGE network, with the features selected by the CFS method, gave 0.649 (95%CI: (0.488, 0.782)) for sensitivity, 0.75 (95%CI: (0.661, 0.822)) for specificity, 0.724 (95%CI: (0.651, 0.797)) for accuracy, 0.753 (95%CI: (0.674, 0.832)) for AUC, 0.603 (95%CI: (0.534, 0.668)) for F_1_-score and 0.471 (95%CI: (0.341, 0.605)) for precision. The other machine learning models achieved AUCs ranging from 0.501 to 0.572.

## 4. Discussion

Common fundus examinations only focus on finding pathological patterns, such as micro-aneurysms, exudates and edema, etc. [7]. It is almost impossible for ophthalmologists to quantitatively measure the various properties of the blood vessels, as there are no such metrics. Therefore, artificial intelligence and machine learning techniques are essential to access these parameters for disease prognostics. In this study, we studied the association between the changes in the retinal vasculature and the changes in the coronary vasculature. The retinal vasculature was manually annotated and quantitatively measured by 96 vascular biomarkers. The stenosis severity of the coronary artery was assessed by the CAD-RADS score for a standardized CAD reporting system. We attempted to use fundus vascular features to predict patients’ CAD-RADS scores by using a graph neural network. We divided our dataset in two ways: (1) CAD-RADS ≤ 1 and CAD-RADS ≥ 2 (model 1); (2) CAT = 0 and CAT = 1 (model 2).

In this study, we examined the association between each fundus feature and the CAD-RADS score using multinomial logistic regression analysis. As summarized in Supplementary Table S2, the widths of both arteries and veins were significantly associated with the CAD-RADS (*p* < 0.05). This finding is consistent with other studies carried out among patients with diabetes, cardiovascular diseases and atherosclerosis [3,12,38]. Moreover, we found that the curvatures for both arteries and veins were significantly associated with the CAD-RADS (*p* < 0.05). The bifurcation angle and the fractal dimension showed no significant difference.

The results shown in Table 3 implied that linking the individuals as a graph based on their similarity in terms of age and gender was beneficial to the predictive task. The CAD-RADS classification performances of GNN on CAD-RADS models 1 and 2 outperformed the traditional machine learning classifiers. Our GNN model achieved an AUC of 0.739 (95%CI: (0.675, 0.804)) for image-wise classification, 0.769 (95%CI: (0.708, 0.831)) for subject-wise classification on model 1, and 0.692 (95%CI: 0.608, 0.776) for image-wise classification and 0.742 (95%CI: (0.662, 0.822)) for subject-wise classification on model 2. The classification performance of the GNN model significantly outperformed other traditional machine learning models, including LR, LDA, KNN, Gaussian NB and SVM on model 1 (McNemar test, *p* < 0.05).

In our study population, 78 subjects had both the left and the right eye images included, while 67 subjects (46%) had one eye image excluded due to suboptimal image quality as assessed by ophthalmologists. Therefore, we built our prediction models based on an image-wise level and evaluated the performance on both the image-wise level and subject-wise level. As we can compare the AUC of the GNN model regarding the image-wise and subject-wise classification from Table 3, the AUC of GNN on model 1 was 0.739 (95%: (0.675, 0.804)) for image-wise classification and 0.769 (95%CI: (0.708, 0.831)) for subject-wise classification. For model 2, the AUC of GNN was 0.692 (95%CI: (0.608, 0.775)) for image-wise classification and 0.742 (95%CI: (0.662, 0.822)) for subject-wise classification. Although not statistically significant, it is implied that using the eye images from both eyes tended to yield better predictions for model 2, predicting more severe or established coronary artery disease, as indicated by a higher AUC.

We performed a comparison study on the performance achieved using a one-, two-, three- and four-layer GraphSAGE architecture. The accuracies with 95% CIs were 88.10% (83.10–93.10), 82.50% (76.60–88.40), 75.00% (68.3–81.7) and 73.10% (66.30–80.00), respectively. The *p*-values of the Z-test (mean-test) showed that one-layer architecture was significantly better than three-layers and four-layers (*p* < 0.001). The difference between one layer and two layers was not significant (*p* = 0.35). Since the two-layer and three-layer architectures were commonly used according to the work by Dwivedi et al. [39], we utilized a two-layer architecture in this study.

There are some limitations to our study. First, we compared the diagnostic performance of GNN and traditional machine learning models. The results showed that using a graph-based model outperformed the traditional methods. Due to the small sample size of the study population, we were unable to build a prognostic model with optimal diagnostic performance. Prospective multi-centre studies of large samples will be needed. Therefore, this study is only a proof-of-concept study. Second, we obtained the retinal vasculature by manual annotation using the “ImageJ” software. Since we had a limited number of fundus images, we wanted to include as many blood vessels as possible. We decided to manually annotate the vasculature and focused on developing the pipeline for vascular feature extraction and building the classification models. In the future, when more fundus images are available, a fully automatic blood vessel segmentation and modeling technique will be required for extracting the vascular structure [40,41,42]. Finally, we only included similarity scores based on two types of demographic information (i.e., age and gender). We also tried to incorporate other patient demographic information, such as smoking status, education level and BMI, but the classification results were not significantly improved. This might be due to the small sample size in our study population; thus, introducing more complicated similarity scores did not help improve the GNN model’s performance. Last but not least, in order to simplify the study, the ophthalmologists only labeled four common eye diseases, i.e., tessellated retina, DM-related retinopathy, AMD and pathologic myopia. Other eye diseases, such as hypertension retinopathy, cataracts and glaucoma, were not included in the list of abnormal conditions, and their associations to atherosclerosis were not studied. In our future work, we plan to assess them in a larger population study.

## 5. Conclusions

In this study, we introduced a graph analysis and graph convolutional neural network for the prediction of CAD-RADS by using quantitative retinal vascular biomarkers. The classification performance outperformed traditional machine learning models. The results showed that by linking the subjects via their age and gender, the retinal vasculature is potentially predictive of the stenosis severity in the coronary artery. These data suggested that common pathophysiologic processes may underlie both micro-vasculature and macro-vasculature.

## Figures and Tables

**Table 1:** Study population demographics stratified by CAD-RADS score.

	0		1		2		3		4		5		Model 1				Model 2			
													CAD-RADS * ≤ 1		CAD-RADS ≥ 2		CAT ** = 0		CAT = 1	
Number of participants	55		15		37		20		13		5		70		75		108		37	
	No.	(%)	No.	(%)	No.	(%)	No.	(%)	No.	(%)	No.	(%)	No.	(%)	No.	(%)	No.	(%)	No.	(%)
Gender																				
Male	28	(50.91)	7	(46.67)	19	(51.35)	17	(85.0)	10	(76.92)	3	(60.0)	35	(50.0)	49	(65.33)	57	(52.78)	27	(72.97)
Female	27	(49.09)	8	(53.33)	18	(48.65)	3	(15.0)	3	(23.08)	2	(40.0)	35	(50.0)	26	(34.67)	51	(47.22)	10	(27.03)
Tobacco use																				
Non-smoker	42	(76.36)	11	(73.33)	21	(56.76)	16	(80.0)	7	(53.85)	4	(80.0)	53	(75.71)	48	(64.0)	76	(70.37)	25	(67.57)
Current smoker	3	(5.45)	3	(20.0)	5	(13.51)	2	(10.0)	2	(15.38)	0	(0)	6	(8.57)	9	(12.0)	12	(11.11)	3	(8.11)
Ex-smoker	10	(18.18)	1	(6.67)	11	(29.73)	2	(10.0)	4	(30.77)	1	(20.0)	11	(15.71)	18	(24.0)	20	(18.52)	9	(24.32)
Retinopathy																				
Non-retinopathy	35	(63.64)	10	(66.67)	22	(59.46)	12	(60.0)	6	(46.15)	3	(60.0)	45	(64.29)	43	(57.33)	64	(59.26)	24	(64.86)
Tessellated retina	12	(21.82)	3	(20.0)	9	(24.32)	5	(25.0)	3	(23.08)	1	(20.0)	15	(21.43)	18	(24.0)	27	(25.0)	6	(16.22)
DM-related retinopathy	2	(3.64)	0	(0)	2	(5.41)	1	(5.0)	1	(7.69)	0	(0)	2	(2.86)	4	(5.33)	4	(3.7)	2	(5.41)
AMD	6	(10.91)	2	(13.33)	5	(13.51)	1	(5.0)	2	(15.38)	1	(20.0)	8	(11.43)	9	(12.0)	13	(12.04)	4	(10.81)
Pathologic myopia	1	(1.82)	0	(0)	2	(5.41)	1	(5.0)	0	(0)	0	(0)	1	(1.43)	3	(4.0)	4	(3.7)	0	(0)
Comorbidities																				
Heart failure	2	(3.64)	1	(6.67)	1	(2.7)	2	(10)	1	(7.69)	0	(0)	3	(4.29)	4	(5.33)	5	(4.63)	2	(5.41)
Ischemic heart disease	12	(21.82)	3	(20)	5	(13.51)	8	(40)	2	(15.38)	1	(20)	15	(21.43)	16	(21.33)	10	(9.26)	21	(56.76)
Hyperlipidemia	17	(30.91)	10	(66.67)	15	(40.54)	15	(75)	8	(61.54)	4	(80)	27	(38.57)	42	(56)	40	(37.04)	29	(78.97)
Hypertension	25	(45.45)	7	(46.67)	18	(48.65)	10	(50)	10	(76.92)	4	(80)	32	(45.71)	42	(56)	47	(43.52)	27	(72.97)
Diabetes mellitus	8	(14.55)	2	(13.33)	2	(5.41)	9	(45)	3	(23.08)	1	(20)	10	(14.29)	15	(20)	15	(13.89)	10	(27.03)
	mean ± std		mean ± std		mean ± std		mean ± std		mean ± std		mean ± std		mean ± std		mean ± std		mean ± std		mean ± std	
Age	54.35 ± 12.33		59.73 ± 10.31		62.86 ± 12.3		61.25 ± 12.51		65.0 ± 8.56		59.2 ± 12.3		55.5 ± 12.06		62.56 ± 11.67		58.48 ± 12.92		61.11 ± 10.37	
BMI (kg/m^2^)	24.52 ± 5.5		25.38 ± 3.16		26.04 ± 4.51		25.68 ± 5.37		25.07 ± 3.14		25.72 ± 1.91		24.7 ± 5.08		25.75 ± 4.38		25.35 ± 5.19		24.94 ± 3.14	
Blood pressure (mmHg)																				
Systolic	129.31 ± 19.83		134.8 ± 16.89		135.11 ± 18.47		123.65 ± 16.5		134.69 ± 17.95		130.2 ± 19.51		130.49 ± 19.26		131.65 ± 18.27		131.16 ± 18.54		130.89 ± 19.41	
Diastolic	79.73 ± 13.38		80.47 ± 10.6		81.72 ± 10.52		75.85 ± 10.44		81.62 ± 11.12		79.4 ± 6.58		79.89 ± 12.77		79.98 ± 10.53		79.69 ± 11.7		80.65 ± 11.53	
Heart rate (BPM)	74.82 ± 11.46		70.27 ± 14.		71.11 ± 10.56		71.3 ± 12.84		68.23 ± 6.02		71.8 ± 18.47		73.84 ± 12.09		70.71 ± 11.05		73.12 ± 11.83		69.59 ± 10.75	

* CAD-RADS: The Coronary Artery Disease Reporting and Data System. ** CAT: The significant CAD-RADS score.

**Table 2:** CAD-RADS prediction models and eye disease.

	CAD-RADS Model 1				CAD-RADS Model 2			
	CAD-RADS ≤ 1	CAD-RADS ≥ 2			CAT = 0	CAT = 1		
Tessellated retina		OR	95%CI	*p*-value		OR	95%CI	*p*-value
OR-Model 1 *	1.00	2.139	(0.188, 24.345)	0.54	1.00	-	(-, -)	-
OR-Model 2 ^†^	1.00	2.257	(0.182, 27.949)	0.526	1.00	-	(-, -)	-
DM-related retinopathy								
OR-Model 1	1.00	1.481	(0.24, 9.119)	0.672	1.00	1.64	(0.249, 10.805)	0.607
OR-Model 2	1.00	2.112	(0.3, 14.881)	0.453	1.00	1.542	(0.205, 11.594)	0.674
AMD								
OR-Model 1	1.00	1.09	(0.45, 2.636)	0.849	1.00	0.628	(0.225, 1.753)	0.375
OR-Model 2	1.00	1.361	(0.524, 3.532)	0.527	1.00	0.733	(0.245, 2.193)	0.578
Pathologic myopia								
OR-Model 1	1.00	1.02	(0.34, 3.057)	0.972	1.00	1.006	(0.284, 3.561)	0.993
OR-Model 2	1.00	1.071	(0.33, 3.476)	0.909	1.00	1.334	(0.344, 5.169)	0.677

* Model 1: adjusted for age, gender. ^†^ Model 2: adjusted for the variables in model 1 plus cardiovascular disease risk factors including systolic blood pressure, heart rate, diabetes (self-reported), BMI and smoking status.

**Table 3:** The classification performance of GraphSAGE and other machine learning models on the CAD-RADS model 1 and model 2, in terms of image-wise and subject-wise classification. Bolded values indicate the best performance across the models.

Methods **	Feature Selection	Sens.	Spec.	Accu.	AUC	F_1_-Score	Precision	*p*-Value *
CAD-RADS Model 1 (class 0: CAD-RADS ≤ 1; class 1: CAD-RADS ≥ 2) for image-wise classification								
GraphSAGE	all	**0.711 (0.621, 0.786)**	0.697 (0.605, 0.776)	**0.704 (0.644, 0.764)**	**0.739 (0.675, 0.804)**	**0.711 (0.672, 0.746)**	**0.711 (0.621, 0.786)**	-
LR	CFS	0.509 (0.418, 0.599)	0.541 (0.448, 0.632)	0.525 (0.459, 0.59)	0.521 (0.445, 0.596)	0.514 (0.473, 0.555)	0.537 (0.443, 0.628)	<0.01
LDA	DISR	0.553 (0.461, 0.641)	0.468 (0.377, 0.561)	0.511 (0.446, 0.577)	0.507 (0.431, 0.583)	0.546 (0.505, 0.586)	0.521 (0.432, 0.608)	<0.05
KNN	CFS	0.158 (0.102, 0.236)	**0.862 (0.785, 0.915)**	0.502 (0.437, 0.568)	0.527 (0.451, 0.603)	0.184 (0.152, 0.221)	0.545 (0.38, 0.702)	<0.01
NB	CFS	0.491 (0.401, 0.582)	0.495 (0.403, 0.588)	0.493 (0.428, 0.559)	0.52 (0.444, 0.596)	0.494 (0.453, 0.535)	0.505 (0.413, 0.596)	<0.01
SVM	all	0.535 (0.444, 0.624)	0.569 (0.475, 0.658)	0.552 (0.486, 0.617)	0.604 (0.53, 0.678)	0.541 (0.5, 0.581)	0.565 (0.471, 0.654)	<0.01
CAD-RADS Model 1 (class 0: CAD-RADS ≤ 1; class 1: CAD-RADS ≥ 2) for subject-wise classification								
GraphSAGE	LAP	**0.747 (0.638, 0.831)**	0.571 (0.455, 0.681)	**0.662 (0.585, 0.739)**	**0.769 (0.708, 0.831)**	**0.725 (0.679, 0.768)**	**0.651 (0.546, 0.743)**	-
LR	CFS	0.507 (0.396, 0.617)	0.543 (0.427, 0.654)	0.524 (0.443, 0.605)	0.512 (0.436, 0.588)	0.514 (0.463, 0.564)	0.543 (0.427, 0.654)	< 0.01
LDA	DISR	0.453 (0.346, 0.566)	0.5 (0.386, 0.614)	0.476 (0.395, 0.557)	0.526 (0.45, 0.601)	0.461 (0.411, 0.512)	0.493 (0.378, 0.608)	<0.05
KNN	CFS	0.387 (0.285, 0.5)	**0.657 (0.54, 0.758)**	0.517 (0.436, 0.599)	0.531 (0.455, 0.607)	0.411 (0.361, 0.463)	0.547 (0.415, 0.673)	<0.01
NB	CFS	0.453 (0.346, 0.566)	0.514 (0.4, 0.628)	0.483 (0.401, 0.564)	0.492 (0.416, 0.568)	0.462 (0.412, 0.513)	0.5 (0.384, 0.616)	<0.01
SVM	SVMB	0.653 (0.541, 0.751)	0.614 (0.497, 0.72)	0.634 (0.556, 0.713)	0.697 (0.629, 0.765)	0.652 (0.602, 0.698)	0.645 (0.533, 0.743)	<0.05
CAD-RADS Model 2 (class 0: CAT = 0; class 1: CAT = 1) for image-wise classification								
GraphSAGE	all	0.544 (0.416, 0.666)	**0.681 (0.606, 0.747)**	0.646 (0.583, 0.709)	**0.692 (0.608, 0.776)**	**0.497 (0.442, 0.552)**	**0.369 (0.274, 0.476)**	-
LR	CFS	**0.561 (0.433, 0.682)**	0.5 (0.425, 0.575)	0.516 (0.45, 0.581)	0.513 (0.426, 0.601)	0.466 (0.414, 0.519)	0.278 (0.205, 0.366)	>0.05
LDA	CFS	0.544 (0.416, 0.666)	0.428 (0.355, 0.504)	0.457 (0.392, 0.523)	0.497 (0.41, 0.584)	0.438 (0.387, 0.49)	0.246 (0.179, 0.328)	>0.05
KNN	CFS	0.228 (0.138, 0.352)	0.819 (0.754, 0.87)	**0.668 (0.606, 0.73)**	0.561 (0.473, 0.649)	0.24 (0.193, 0.294)	0.302 (0.186, 0.451)	>0.05
NB	LAP	0.544 (0.416, 0.666)	0.422 (0.349, 0.498)	0.453 (0.388, 0.518)	0.498 (0.411, 0.585)	0.437 (0.386, 0.489)	0.244 (0.178, 0.326)	>0.05
SVM	LAP	0.544 (0.416, 0.666)	0.488 (0.413, 0.563)	0.502 (0.437, 0.568)	0.514 (0.426, 0.601)	0.451 (0.399, 0.503)	0.267 (0.195, 0.354)	>0.05
CAD-RADS Model 2 (class 0: CAT = 0; class 1: CAT = 1) for subject-wise classification								
GraphSAGE	CFS	**0.649 (0.488, 782)**	0.75 (0.661, 0.822)	**0.724 (0.651, 0.797)**	**0.753 (0.674, 0.832)**	**0.603 (0.534, 0.668)**	**0.471 (0.341, 0.605)**	-
LR	CFS	0.568 (0.409, 0.713)	0.444 (0.354, 0.538)	0.476 (0.395, 0.557)	0.501 (0.414, 0.588)	0.459 (0.395, 0.523)	0.259 (0.176, 0.364)	>0.05
LDA	CFS	0.541 (0.384, 0.69)	0.463 (0.372, 0.557)	0.483 (0.401, 0.564)	0.501 (0.414, 0.588)	0.442 (0.379, 0.508)	0.256 (0.173, 0.363)	>0.05
KNN	CFS	0.243 (0.134, 0.401)	**0.759 (0.671, 0.83)**	0.628 (0.549, 0.706)	0.572 (0.485, 0.66)	0.246 (0.189, 0.313)	0.257 (0.142, 0.421)	>0.05
NB	CMIM	0.568 (0.409, 0.713)	0.417 (0.328, 0.511)	0.455 (0.374, 0.536)	0.52 (0.432, 0.607)	0.453 (0.39, 0.517)	0.25 (0.17, 0.352)	<0.05
SVM	SVMB	0.595 (0.435, 0.737)	0.556 (0.462, 0.646)	0.566 (0.485, 0.646)	0.565 (0.477, 0.653)	0.505 (0.439, 0.57)	0.314 (0.218, 0.43)	>0.05

* *p*-value based on McNemar’s testing. ** Abbreviations: GraphSAGE—the graph sample and aggregate network; LR—logistic regression classifier; LDA: linear discrimation analysis classifier; KNN- K-nearest neightbour classifier; NB—naive Bayesian classifer; SVM—support vector machine classifier.

## Data Availability

The datasets used in this current study are available from the corresponding author on reasonable request.

## Supplementary Materials

The following supporting information can be downloaded at: https://www.mdpi.com/article/10.3390/diagnostics12061390/s1, Supplementary document. Figure S1: The CCTA examples for the subjects of diagnosed CADRADS = 1 (minimal), 2 (mild occlusion), 3 (moderate), 4 (severe) and 5 (occluded), respectively; Table S1: The Odd-ratio between CAD-RADS and 4 common eye diseases; Table S2: The odd-ratio between the CAD-RADS and each retinal vascular biomarker. Bolded values indicated the significant association (*p* < 0.05).

## Appendix A

Let *n_i_*: *n_j_* be the two nodes representing two images of patients, *g_i_*, *g_j_* be their genders, and *a_i_*, *a_j_* be their ages, the similarity score (denoted as *SimScore*) is calculated by:

$$SimScore\left(n_{i},\;n_{j}\right)=Diff\left(g_{i},g_{j}\right)+Diff\left(a_{i},a_{j}\right),$$

where

$$Diff\left(g_{i},g_{j}\right)=\{\begin{array}{cc} 1, & if\;g_{i}=g_{j}\\ 0, & if\;g_{i}\neq g_{j} \end{array}$$

$$Diff\left(a_{i},a_{j}\right)=\{\begin{array}{cc} 1, & if\;\left|a_{i}-a_{j}\right|\leq 5\\ 0, & if\;\left|a_{i}-a_{j}\right|>5. \end{array}$$

The value of $SimScore\left(n_{i},\;n_{j}\right)$ could be either 0, 1 or 2, thus $SimScore\left(n_{i},\;n_{j}\right)>0$ results in an edge between nodes *n_i_* and *n_j_*. A larger $SimScore\left(n_{i},\;n_{j}\right)$ indicated a stronger connection between the two nodes.

## Appendix B

In this study: we built a GraphSAGE network with two SAGEConv layers with the mean pooling aggregator. The network architecture is summarized in the Table A1 below.

**Table A1:** The GraphSAGE network architecture used in this study, which was a two-layer network with one dropout layer.

Layers	Input Features	Output Features	Parameters
Input = *G*	96	-	-
SAGEConv	96	128	Aggregator = mean
ReLU	-	-	-
Dropout layers	-	-	Probability = 0.5
SAGEConv	128	2	Aggregator = mean
Softmax layer	2	2	-
Loss	-	-	Cross-entropy loss
