# Supplementary material for: Predicting CT-Based Coronary Artery Disease Using Vascular Biomarkers Derived from Fundus Photographs with a Graph Convolutional Neural Network
Source: Diagnostics (Basel). 2022 Jun 4;12(6):1390. doi: 10.3390/diagnostics12061390 (PMC9221688; doi:10.3390/diagnostics12061390)
Supplement: Supplementary file 1 [file diagnostics-12-01390-s001.zip › diagnostics-1667574-supplementary.pdf]

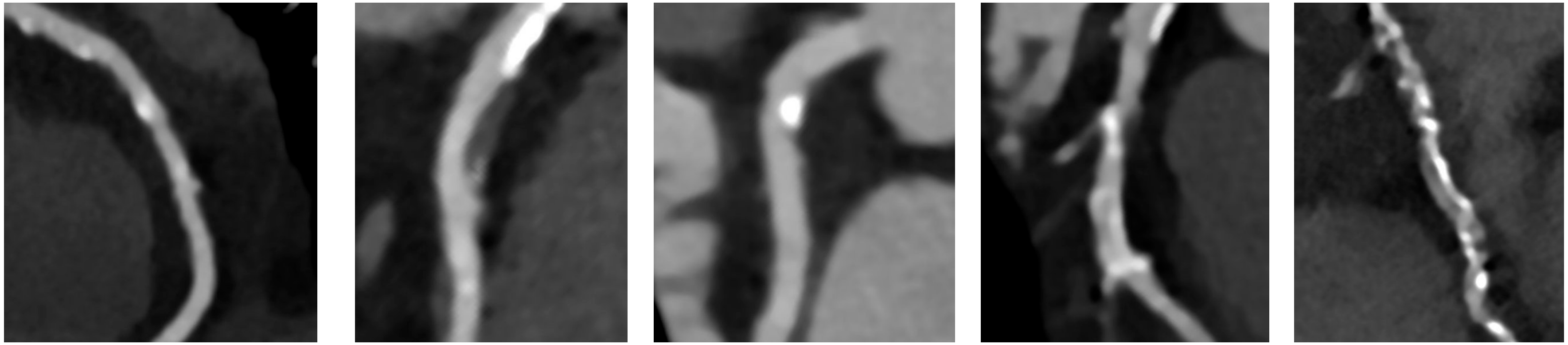

CADRADS = 1 (minimal occlusion) | CADRADS = 2 (mild occlusion) | CADRADS = 3 (moderate occlusion) | CADRADS = 4 (severe occlusion) | CADRADS = 5 (occluded)

Figure S1. The CCTA examples for the subjects of diagnosed CADRADS=1 (minimal), 2 (mild occlusion), 3 (moderate), 4 (severe) and 5 (occluded), respectively.

Table S1. The Odd-ratio between CAD-RADS and 4 common eye diseases.

	0		1			2			3			4			5	
		OR	95% CI	P-value	OR	95% CI	P-value	OR	95% CI	P-value	OR	95% CI	P-value	OR	95% CI	P-value
Tessellated retina																
OR-Model 1*	1.00	-	(-, -)	-	1.82	(0.131,25.358)	0.656	3.646	(0.142,93.665)	0.435	-	(-, -)	-	-	(-, -)	-
OR-Model 2†	1.00	-	(-, -)	-	2.127	(0.14,32.272)	0.586	2.711	(0.065,113.66)	0.601	-	(-, -)	-	-	(-, -)	-
DM-related retinopathy																
OR-Model 1	1.00	-	(-, -)	-	0.973	(0.112,8.458)	0.98	1.248	(0.095,16.406)	0.866	2.256	(0.155,32.818)	0.551	-	(-, -)	-
OR-Model 2	1.00	-	(-, -)	-	1.542	(0.132,18.051)	0.73	1.222	(0.063,23.571)	0.895	4.257	(0.204,88.804)	0.35	-	(-, -)	-
AMD																
OR-Model 1	1.00	0.964	(0.223,4.166)	0.961	1.066	(0.35,3.253)	0.91	1.04	(0.266,4.065)	0.955	1.171	(0.236,5.819)	0.847	1.058	(0.102,10.959)	0.962
OR-Model 2	1.00	1.139	(0.232,5.594)	0.873	1.04	(0.31,3.485)	0.949	2.573	(0.529,12.529)	0.242	1.929	(0.312,11.933)	0.48	1.196	(0.101,14.152)	0.887
Pathologic myopia																

OR-Model 1	1.00	0.973	(0.163,5.809)	0.976	0.977	(0.25,3.812)	0.973	0.507	(0.051,5.031)	0.562	1.706	(0.255,11.405)	0.582	1.939	(0.164,22.89)	0.599
OR-Model 2	1.00	1.045	(0.154,7.11)	0.964	0.86	(0.196,3.778)	0.841	0.804	(0.065,9.905)	0.865	3.456	(0.387,30.84)	0.267	1.853	(0.126,27.211)	0.653

* Model 1: adjusted for age, gender.

† Model 2: adjusted for the variables in model 1 plus cardiovascular disease risk factor including systolic blood pressure, heart rate, diabetes (self-reported),BMI and smoking status.

Table S2. The odd-ratio between the CAD-RADS and each retinal vascular biomarker. Bolded values indicated the significant association ($p < 0.05$).

		CAD-RADS Model 1				CAD-RADS Model 2		
		CAD-RADS<=1	CAD-RADS>=2			CAT=0	CAT=1	
			OR (95%CI)	P-values			OR (95%CI)	P-values
Mean arterial width	OR Model 1	1.00	0 (0, +∞)	0.49		1.00	+∞ (0, +∞)	0.58
	OR Model 2	1.00	0 (0, +∞)	0.10		1.00	0 (0, +∞)	0.89
Std arterial width	OR Model 1	1.00	0 (0, 0.68)	**<0.05**		1.00	0 (0, +∞)	0.10
	OR Model 2	1.00	0 (0, 1.974)	0.06		1.00	0 (0, +∞)	0.27
Med arterial width	OR Model 1	1.00	0.658 (0, +∞)	0.94		1.00	0.002(0, +∞)	0.87
	OR Model 2	1.00	+∞ (0, +∞)	0.28		1.00	+∞ (0, +∞)	0.97
Min arterial width	OR Model 1	1.00	0.338 (0, +∞)	0.83		1.00	0 (0, +∞)	0.29
	OR Model 2	1.00	64.053 (0, +∞)	0.50		1.00	0 (0, +∞)	0.17
Max arterial width	OR Model 1	1.00	+∞ (0, +∞)	0.43		1.00	0 (0, +∞)	0.46
	OR Model 2	1.00	+∞ (0, +∞)	0.48		1.00	+∞ (0, +∞)	0.48
Mean venous width	OR Model 1	1.00	0.002 (0, +∞)	0.40		1.00	0 (0, 1.208)	0.05
	OR Model 2	1.00	0 (0, +∞)	0.22		1.00	0 (0, +∞)	0.35
Std venous width	OR Model 1	1.00	0.013 (0, 18.353)	0.24		1.00	+∞ (0, +∞)	0.76
	OR Model 2	1.00	0 (0, 1.653)	0.06		1.00	+∞ (0, +∞)	0.38
Med venous width	OR Model 1	1.00	83.730 (0.002, +∞)	0.41		1.00	+∞ (0, +∞)	0.06
	OR Model 2	1.00	+∞ (0.013, +∞)	0.19		1.00	+∞ (0, +∞)	0.23
Min venous width	OR Model 1	1.00	+∞ (0.251, +∞)	0.12		1.00	+∞ (0, +∞)	0.11
	OR Model 2	1.00	+∞ (0.241, +∞)	0.11		1.00	+∞ (0, +∞)	0.23

Max venous width	OR Model 1	1.00	+∞ (0.033, +∞)	0.21		1.00	+∞ (+∞, +∞)	**<0.05**
	OR Model 2	1.00	+∞ (0.419, +∞)	0.07		1.00	+∞ (0, +∞)	0.67
Mean arτerial angle	OR Model 1	1.00	+∞ (0, +∞)	0.32		1.00	0 (0, +∞)	0.07
	OR Model 2	1.00	+∞ (0, +∞)	0.13		1.00	0 (0, +∞)	0.80
Std arterial angle	OR Model 1	1.00	+∞ (+∞, +∞)	**<0.05**		1.00	0 (0, 81.993)	0.06
	OR Model 2	1.00	+∞ (+∞, +∞)	**<0.05**		1.00	0 (0, +∞)	0.79
Med arterial angle	OR Model 1	1.00	0.002 (0, +∞)	0.47		1.00	+∞ (0, +∞)	0.08
	OR Model 2	1.00	0 (0, +∞)	0.24		1.00	0 (0, +∞)	0.96
Min arterial angle	OR Model 1	1.00	+∞ (0.187, +∞)	0.08		1.00	0.011 (0, +∞)	0.77
	OR Model 2	1.00	+∞ (0.018, +∞)	0.13		1.00	+∞ (0, +∞)	0.51
Max arterial angle	OR Model 1	1.00	0 (0, 0.025)	**<0.05**		1.00	+∞ (0, +∞)	0.12
	OR Model 2	1.00	0 (0, 0.003)	**<0.05**		1.00	+∞ (0, +∞)	0.94
Mean venous angle	OR Model 1	1.00	+∞ (0, +∞)	0.22		1.00	+∞ (0, +∞)	0.15
	OR Model 2	1.00	+∞ (0.009, +∞)	0.12		1.00	+∞ (0, +∞)	0.89
Std venous angle	OR Model 1	1.00	0 (0, 0.003)	**<0.05**		1.00	0.023(0, +∞)	0.82
	OR Model 2	1.00	0 (0, 0.005)	**<0.05**		1.00	0 (0, +∞)	0.56
Med venous angle	OR Model 1	1.00	0.237 (0, +∞)	0.82		1.00	0 (0, +∞)	0.56
	OR Model 2	1.00	0.020 (0, +∞)	0.59		1.00	0 (0, +∞)	0.98
Min venous angle	OR Model 1	1.00	0 (0, 0.077)	**<0.05**		1.00	+∞ (0.006, +∞)	0.08
	OR Model 2	1.00	0 (0, 0.213)	**<0.05**		1.00	4.149 (0, +∞)	1.00
Max venous angle	OR Model 1	1.00	+∞ (0.119, +∞)	0.14		1.00	+∞ (0, +∞)	0.20
	OR Model 2	1.00	+∞ (0.008, +∞)	0.33		1.00	+∞ (0, +∞)	0.57
Mean τ1	OR Model 1	1.00	0 (0, +∞)	0.75		1.00	+∞ (+∞, +∞)	**<0.05**
	OR Model 2	1.00	0 (0, +∞)	0.91		1.00	+∞ (0, +∞)	0.85
Sτd τ1	OR Model 1	1.00	0 (0, 0)	**<0.05**		1.00	0 (0, 0)	**<0.05**
	OR Model 2	1.00	0 (0, 0)	**<0.05**		1.00	+∞ (0, +∞)	0.90
Med τ1	OR Model 1	1.00	+∞ (0, +∞)	0.46		1.00	0 (0, +∞)	0.14

	OR Model 2	1.00	+∞ (0, +∞)	0.24		1.00	+∞ (0, +∞)	0.90
Min τ1	OR Model 1	1.00	+∞ (1.611, +∞)	**<0.05**		1.00	+∞ (2.093, +∞)	**<0.05**
	OR Model 2	1.00	+∞ (0.611, +∞)	0.06		1.00	+∞ (0, +∞)	0.56
Max τ1	OR Model 1	1.00	+∞ (+∞, +∞)	**<0.05**		1.00	+∞ (0, +∞)	0.32
	OR Model 2	1.00	+∞ (+∞, +∞)	**<0.05**		1.00	0 (0, +∞)	0.70
Mean τ2	OR Model 1	1.00	0 (0, 0)	**<0.05**		1.00	0 (0, 0)	**<0.05**
	OR Model 2	1.00	0 (0, 0)	**<0.05**		1.00	0 (0, 0)	**<0.05**
Std τ2	OR Model 1	1.00	0 (0, 0)	**<0.05**		1.00	0 (0, 0)	**<0.05**
	OR Model 2	1.00	0 (0, 0)	**<0.05**		1.00	0 (0, 0)	**<0.05**
Med τ2	OR Model 1	1.00	0 (0, 0)	**<0.05**		1.00	0 (0, 0)	**<0.05**
	OR Model 2	1.00	0 (0, 0)	**<0.05**		1.00	0 (0, 0)	**<0.05**
Min τ2	OR Model 1	1.00	0 (0, 0)	**<0.05**		1.00	0 (0, 0)	**<0.05**
	OR Model 2	1.00	0 (0, 0)	**<0.05**		1.00	0 (0, 0)	**<0.05**
Max τ2	OR Model 1	1.00	0 (0, 0)	**<0.05**		1.00	0 (0, 0)	**<0.05**
	OR Model 2	1.00	0 (0, 0)	**<0.05**		1.00	0 (0, 0)	**<0.05**
Mean τ3	OR Model 1	1.00	+∞ (0, +∞)	0.25		1.00	+∞ (0, +∞)	0.12
	OR Model 2	1.00	+∞ (0, +∞)	0.10		1.00	0 (0, +∞)	0.95
Std τ3	OR Model 1	1.00	0 (0, 0)	**<0.05**		1.00	0 (0, +∞)	0.24
	OR Model 2	1.00	0 (0, 0)	**<0.05**		1.00	0 (0, +∞)	0.42
Med τ3	OR Model 1	1.00	0 (0, 0)	**<0.05**		1.00	0 (0, +∞)	0.11
	OR Model 2	1.00	0 (0, 0)	**<0.05**		1.00	0 (0, +∞)	0.06
Min τ3	OR Model 1	1.00	26.828 (0, +∞)	0.83		1.00	0 (0, +∞)	0.60
	OR Model 2	1.00	+∞ (0, +∞)	0.46		1.00	0 (0, +∞)	0.18
Max τ3	OR Model 1	1.00	+∞ (+∞, +∞)	**<0.05**		1.00	+∞ (0, +∞)	0.48
	OR Model 2	1.00	+∞ (+∞, +∞)	**<0.05**		1.00	+∞ (0, +∞)	0.50
Mean τ4	OR Model 1	1.00	0 (0, +∞)	0.50		1.00	0 (0, +∞)	0.22
	OR Model 2	1.00	0 (0, +∞)	0.19		1.00	+∞ (0, +∞)	0.69

Std τ4	OR Model 1	1.00	+∞ (+∞, +∞)	**<0.05**		1.00	+∞ (0, +∞)	0.57
	OR Model 2	1.00	+∞ (+∞, +∞)	**<0.05**		1.00	0 (0, +∞)	0.69
Med τ4	OR Model 1	1.00	+∞ (0.264, +∞)	0.06		1.00	0 (0, +∞)	0.44
	OR Model 2	1.00	+∞ (0.481, +∞)	0.06		1.00	+∞ (0, +∞)	0.76
Min τ4	OR Model 1	1.00	0.0115 (0, +∞)	0.80		1.00	+∞ (0, +∞)	0.33
	OR Model 2	1.00	0 (0, +∞)	0.44		1.00	+∞ (0, +∞)	0.28
Max τ4	OR Model 1	1.00	0 (0, 0)	**<0.05**		1.00	+∞ (0, +∞)	0.87
	OR Model 2	1.00	0 (0, 0)	**<0.05**		1.00	+∞ (0, +∞)	0.61
Mean τ5	OR Model 1	1.00	0 (0, +∞)	0.56		1.00	+∞ (0, +∞)	0.10
	OR Model 2	1.00	0 (0, +∞)	0.30		1.00	0 (0, +∞)	0.64
Std τ5	OR Model 1	1.00	+∞ (0, +∞)	0.22		1.00	+∞ (0, +∞)	0.16
	OR Model 2	1.00	+∞ (0, +∞)	0.23		1.00	+∞ (0, +∞)	0.60
Med τ5	OR Model 1	1.00	0 (0, 38.383)	0.09		1.00	0 (0, +∞)	0.08
	OR Model 2	1.00	0 (0, 0.006)	**<0.05**		1.00	0 (0, +∞)	0.65
Min τ5	OR Model 1	1.00	+∞ (0, +∞)	0.45		1.00	+∞ (0, +∞)	0.36
	OR Model 2	1.00	+∞ (0, +∞)	0.77		1.00	+∞ (0, +∞)	0.49
Max τ5	OR Model 1	1.00	0.002 (0, +∞)	0.65		1.00	0 (0, +∞)	0.08
	OR Model 2	1.00	0.0118(0, +∞)	0.80		1.00	0 (0, +∞)	0.89
Mean τ6	OR Model 1	1.00	0 (0, +∞)	0.41		1.00	0 (0, +∞)	0.09
	OR Model 2	1.00	0 (0, +∞)	0.63		1.00	+∞ (0, +∞)	0.96
Std τ6	OR Model 1	1.00	+∞ (0, +∞)	0.45		1.00	+∞ (+∞, +∞)	**<0.05**
	OR Model 2	1.00	+∞ (0, +∞)	0.54		1.00	+∞ (0, +∞)	0.19
Med τ6	OR Model 1	1.00	+∞ (0, +∞)	0.62		1.00	+∞ (+∞, +∞)	**<0.05**
	OR Model 2	1.00	+∞ (0, +∞)	0.46		1.00	+∞ (0, +∞)	0.16
Min τ6	OR Model 1	1.00	+∞ (0, +∞)	0.31		1.00	0 (0, +∞)	0.95
	OR Model 2	1.00	+∞ (0, +∞)	0.15		1.00	0 (0, +∞)	0.91
Max τ6	OR Model 1	1.00	0 (0, +∞)	0.12		1.00	0 (0, 0)	**<0.05**

	OR Model 2	1.00	0 (0, +∞)	0.13		1.00	0 (0, +∞)	0.21
Mean τ7	OR Model 1	1.00	0 (0, +∞)	0.93		1.00	0 (0, +∞)	0.06
	OR Model 2	1.00	+∞ (0, +∞)	0.97		1.00	+∞ (0, +∞)	0.58
Std τ7	OR Model 1	1.00	0 (0, +∞)	0.31		1.00	0 (0, +∞)	0.54
	OR Model 2	1.00	0 (0, +∞)	0.41		1.00	0 (0, +∞)	0.67
Med τ7	OR Model 1	1.00	+∞ (+∞, +∞)	**<0.05**		1.00	+∞ (0, +∞)	0.18
	OR Model 2	1.00	+∞ (+∞, +∞)	**<0.05**		1.00	+∞ (0, +∞)	0.80
Min τ7	OR Model 1	1.00	0 (0, +∞)	0.49		1.00	0 (0, +∞)	0.57
	OR Model 2	1.00	0 (0, +∞)	0.76		1.00	0 (0, +∞)	0.51
Max τ7	OR Model 1	1.00	+∞ (0, +∞)	0.71		1.00	+∞ (0, +∞)	0.09
	OR Model 2	1.00	0 (0, +∞)	0.94		1.00	+∞ (0, +∞)	0.78
Mean τ8	OR Model 1	1.00	+∞ (0, +∞)	0.15		1.00	+∞ (0, +∞)	0.14
	OR Model 2	1.00	+∞ (0, +∞)	0.19		1.00	0 (0, +∞)	0.55
Std τ8	OR Model 1	1.00	0 (0, +∞)	0.35		1.00	0 (0, +∞)	0.05
	OR Model 2	1.00	0 (0, +∞)	0.26		1.00	+∞ (0, +∞)	0.97
Med τ8	OR Model 1	1.00	0 (0, +∞)	0.58		1.00	0 (0, +∞)	0.07
	OR Model 2	1.00	0 (0, +∞)	0.36		1.00	0 (0, +∞)	0.67
Min τ8	OR Model 1	1.00	0 (0, +∞)	0.27		1.00	0 (0, +∞)	0.70
	OR Model 2	1.00	0 (0, +∞)	0.14		1.00	+∞ (0, +∞)	0.94
Max τ8	OR Model 1	1.00	+∞ (0, +∞)	0.30		1.00	+∞ (0, +∞)	0.05
	OR Model 2	1.00	+∞ (0, +∞)	0.18		1.00	+∞ (0, +∞)	0.77
Mean τ9	OR Model 1	1.00	0 (0, +∞)	0.69		1.00	0 (0, 0)	**<0.05**
	OR Model 2	1.00	0 (0, +∞)	0.17		1.00	0 (0, +∞)	0.14
Std τ9	OR Model 1	1.00	+∞ (0, +∞)	0.15		1.00	+∞ (+∞, +∞)	**<0.05**
	OR Model 2	1.00	+∞ (31.392, +∞)	**<0.05**		1.00	+∞ (+∞, +∞)	**<0.05**
Med τ9	OR Model 1	1.00	0 (0, 54.828)	0.16		1.00	+∞ (0.002, +∞)	0.08
	OR Model 2	1.00	0 (0, +∞)	0.19		1.00	+∞ (0, +∞)	0.38

Min τ9	OR Model 1	1.00	+∞ (0.322, +∞)	0.11		1.00	+∞ (0.002, +∞)	0.15
	OR Model 2	1.00	+∞ (0.255, +∞)	0.10		1.00	+∞ (0, +∞)	0.60
Max τ9	OR Model 1	1.00	+∞ (0, +∞)	0.50		1.00	0 (0, +∞)	0.07
	OR Model 2	1.00	5.371(0, +∞)	0.85		1.00	0 (0, +∞)	0.18
Mean τ10	OR Model 1	1.00	0 (0, +∞)	0.11		1.00	0 (0, +∞)	0.18
	OR Model 2	1.00	0 (0, +∞)	0.09		1.00	0 (0, +∞)	0.70
Std τ10	OR Model 1	1.00	+∞ (0, +∞)	0.11		1.00	0 (0, +∞)	0.37
	OR Model 2	1.00	+∞ (0, +∞)	0.12		1.00	0 (0, +∞)	0.95
Med τ10	OR Model 1	1.00	+∞ (12.0915, +∞)	**<0.05**		1.00	+∞ (1.878, +∞)	**<0.05**
	OR Model 2	1.00	+∞ (+∞, +∞)	**<0.05**		1.00	+∞ (0, +∞)	0.14
Min τ10	OR Model 1	1.00	+∞ (1.302, +∞)	**<0.05**		1.00	+∞ (0, +∞)	0.19
	OR Model 2	1.00	+∞ (1.232, +∞)	**<0.05**		1.00	0 (0, +∞)	0.95
Max τ10	OR Model 1	1.00	0 (0, 0.004)	**<0.05**		1.00	0 (0, +∞)	0.70
	OR Model 2	1.00	0 (0, 0.012)	**<0.05**		1.00	0 (0, +∞)	0.87
Mean τ11	OR Model 1	1.00	+∞ (0, +∞)	0.35		1.00	+∞ (+∞, +∞)	**<0.05**
	OR Model 2	1.00	+∞ (0, +∞)	0.08		1.00	+∞ (0, +∞)	0.29
Sτd τ11	OR Model 1	1.00	0 (0, +∞)	0.81		1.00	0 (0, +∞)	0.82
	OR Model 2	1.00	0 (0, +∞)	0.68		1.00	0 (0, +∞)	0.76
Med τ11	OR Model 1	1.00	0 (0, +∞)	0.43		1.00	0 (0, +∞)	0.06
	OR Model 2	1.00	0 (0, +∞)	0.68		1.00	0 (0, +∞)	0.90
Min τ11	OR Model 1	1.00	0 (0, +∞)	0.75		1.00	0 (0, 0)	**<0.05**
	OR Model 2	1.00	+∞ (0, +∞)	0.79		1.00	0 (0, +∞)	0.72
Max τ11	OR Model 1	1.00	0 (0, 0)	**<0.05**		1.00	0 (0, +∞)	0.06
	OR Model 2	1.00	0 (0, 0)	**<0.05**		1.00	0 (0, +∞)	0.83
Mean τ12	OR Model 1	1.00	0 (0, 0)	**<0.05**		1.00	0 (0, +∞)	0.30
	OR Model 2	1.00	0 (0, 0)	**<0.05**		1.00	+∞ (0, +∞)	0.93
Std τ12	OR Model 1	1.00	+∞ (+∞, +∞)	**<0.05**		1.00	+∞ (0, +∞)	0.17

	OR Model 2	1.00	+∞ (+∞, +∞)	**<0.05**		1.00	0 (0, +∞)	0.94
Med τ12	OR Model 1	1.00	+∞ (+∞, +∞)	**<0.05**		1.00	+∞ (0, +∞)	0.06
	OR Model 2	1.00	+∞ (+∞, +∞)	**<0.05**		1.00	+∞ (0, +∞)	0.47
Min τ12	OR Model 1	1.00	0 (0, 0)	**<0.05**		1.00	0 (0, +∞)	0.18
	OR Model 2	1.00	0 (0, 0)	**<0.05**		1.00	0 (0, +∞)	0.93
Max τ12	OR Model 1	1.00	0 (0, +∞)	0.09		1.00	0 (0, +∞)	0.24
	OR Model 2	1.00	0 (0, +∞)	0.10		1.00	+∞ (0, +∞)	0.85
Mean τ13	OR Model 1	1.00	0 (0, +∞)	0.26		1.00	0 (0, 0)	**<0.05**
	OR Model 2	1.00	0 (0, +∞)	0.06		1.00	0 (0, +∞)	0.27
Std τ13	OR Model 1	1.00	+∞ (0, +∞)	0.85		1.00	0 (0, +∞)	0.12
	OR Model 2	1.00	+∞ (0, +∞)	0.70		1.00	0 (0, +∞)	0.91
Med τ13	OR Model 1	1.00	+∞ (0.011, +∞)	0.08		1.00	+∞ (0, +∞)	0.11
	OR Model 2	1.00	+∞ (0.001, +∞)	0.09		1.00	+∞ (0, +∞)	0.84
Min τ13	OR Model 1	1.00	+∞ (0, +∞)	0.80		1.00	+∞ (0.623, +∞)	0.05
	OR Model 2	1.00	0 (0, +∞)	0.73		1.00	+∞ (0, +∞)	0.74
Max τ13	OR Model 1	1.00	+∞ (+∞, +∞)	**<0.05**		1.00	+∞ (0, +∞)	**0.05**
	OR Model 2	1.00	+∞ (+∞, +∞)	**<0.05**		1.00	+∞ (0, +∞)	0.80
Mean τ14	OR Model 1	1.00	+∞ (+∞, +∞)	**<0.05**		1.00	+∞ (0, +∞)	0.32
	OR Model 2	1.00	+∞ (+∞, +∞)	**<0.05**		1.00	0 (0, +∞)	0.93
Std τ14	OR Model 1	1.00	0 (0, 0)	**<0.05**		1.00	0 (0, +∞)	0.25
	OR Model 2	1.00	0 (0, 0)	**<0.05**		1.00	+∞ (0, +∞)	0.90
Med τ14	OR Model 1	1.00	0 (0, 0)	**<0.05**		1.00	0 (0, +∞)	0.06
	OR Model 2	1.00	0 (0, 0)	**<0.05**		1.00	0 (0, +∞)	0.51
Min τ14	OR Model 1	1.00	+∞ (+∞, +∞)	**<0.05**		1.00	+∞ (0, +∞)	0.18
	OR Model 2	1.00	+∞ (+∞, +∞)	**<0.05**		1.00	+∞ (0, +∞)	0.95
Max τ14	OR Model 1	1.00	+∞ (0, +∞)	0.29		1.00	+∞ (0, +∞)	0.52
	OR Model 2	1.00	+∞ (0, +∞)	0.36		1.00	0 (0, +∞)	0.79

Box dimension	OR Model 1	1.00	+∞ (3.423, +∞)	**<0.05**		1.00	+∞ (0.004, +∞)	0.13
	OR Model 2	1.00	+∞ (2.390, +∞)	**<0.05**		1.00	+∞ (0, +∞)	0.55
Information dimension	OR Model 1	1.00	+∞ (3.224, +∞)	**<0.05**		1.00	+∞ (0, +∞)	0.29
	OR Model 2	1.00	+∞ (2.093, +∞)	**<0.05**		1.00	+∞ (0, +∞)	0.59
Correlation dimension	OR Model 1	1.00	+∞ (3.736 +∞)	**<0.05**		1.00	+∞ (0, +∞)	0.14
	OR Model 2	1.00	+∞ (2.410, +∞)	**<0.05**		1.00	+∞ (0, +∞)	0.53
